# Design of Liposomal Lidocaine/Cannabidiol Fixed Combinations for Local Neuropathic Pain Treatment

**DOI:** 10.3390/pharmaceutics14091915

**Published:** 2022-09-10

**Authors:** Silvia Franzè, Liliana Angelo, Antonella Casiraghi, Paola Minghetti, Francesco Cilurzo

**Affiliations:** Department of Pharmaceutical Sciences, University of Milan, Via G. Colombo 71, 20133 Milan, Italy

**Keywords:** cannabidiol, lidocaine, pain management, fixed combination, deformable liposomes

## Abstract

The administration of drug fixed combinations by nanocarriers is a new attractive approach since it can allow improvements in both the skin penetration of cargo compounds and their synergistic effects. The cutaneous administration of lidocaine (LD) and cannabidiol (CBD) combination can be useful for the local treatment of neuropathic pain. In fact, these drugs might exert a complementary effect on pain acting on sodium and calcium channels. In this study, the feasibility to deliver this combination in the deeper layers of the skin using deformable liposomes was studied. Based on a study of the drug affinity for lipid components performed by DSC, CBD was loaded in the lipid bilayer for limiting the leakage, while LD was loaded in the inner core by a pH gradient method (G-liposomes) or after previous encapsulation in micelle (DiMiL). The effect of the presence of Tween 80 in the liposome membrane was also evaluated. DiMiL increased both the skin permeation and the retention in the dermis of CBD and LD with respect to G-liposomes (R_24dermis_: 11.52 ± 2.4 against 4.51 ± 0.8 µg/cm^2^ for CBD; 19.6 ± 2.9 against 3.2 ± 0.1 µg/cm^2^ for LD). Moreover, both DiMiL and G-liposomes were more efficient than control formulations carrying free drugs in improving drug skin permeation. Interestingly, in the presence of a drug exerting a fluidizing effect such as CBD, the removal of Tween 80 from the composition led to an improved control of drug release and a higher extent of drug retention in the dermis layer.

## 1. Introduction

Neuropathic pain has both peripheral and central etiologies including diabetes, chemotherapy-induced peripheral neuropathy, radicular pain, postsurgical chronic neuropathic pain, multiple sclerosis, spinal-cord injury-related pain, and postherpetic neuralgia, to mention a few [1]. This condition severely impacts on the quality of life of patients and is associated with symptoms such as burning, pins and needles (paresthesia), tingling, numbness, electric shocks/shooting, crawling (formication), itching, and intolerance to temperatures. Systemic therapies include antidepressants and anticonvulsant and strong analgesic drugs, such as tramadol, which are obviously associated with severe side effects. Topical analgesics provide pain relief in a variety of neuropathic pain conditions, but this application is still currently limited to the use of lidocaine patches and capsaicin. Nevertheless, the skin can represent a valuable target because of the wide number of sensory receptors distributed in epidermis, dermis, hair follicles, nerves, and hypodermis. Moreover, pain management often requires a combination therapy since about 45% of patients do not respond to a single treatment. In view of this, the presence of multiple receptors in the skin and the decrease in side effects of topically applied analgesic drugs make the administration of drug combination more feasible, and their (trans)dermal administration even more attractive [2].

Besides lidocaine (LD), which exerts the analgesic effect mainly suppressing the activity of peripheral sodium channels, topically applied cannabidiol (CBD) has also been proposed for chronic pain management even if this activity is documented by few clinical data [3].

CBD in fact binds to endocannabinoids receptors that regulate signaling pathway involved in pain and inflammatory management. Those receptors are significantly expressed in the skin tissue, and they are CB1 and CB2 receptors, which can be found in keratinocytes, cutaneous nerve fibers, dermal cells, melanocytes, glands, and follicles, and the Transient Receptor Potential (TRP) Receptors that were found in several skin cells and are involved in the regulation of skin homeostasis and anti-inflammatory responses [3]. For these properties, CBD has been proposed as a good candidate for the treatment of several inflammatory-based skin disorders such as psoriasis, eczema, and atopic dermatitis, etc. [3].

Basing on these considerations, LD and CBD may represent a valuable combination of drugs to be applied on the skin to reach a synergic effect acting on different pathways. Before demonstrating the effectiveness of this association for pain management, however, it is mandatory to overcome issues related to different physico-chemical features of the two compounds that can, in turn, determine a different depth of penetration in the skin. To assure the delivery of both drugs deeply in the skin to target the receptors present in nerve fibers, the loading of the two drugs in a nanovector able to drive the skin penetration can be a proper option. This is an emerging strategy and, to the best of our knowledge, only two papers dealing with the cutaneous administration of fixed combinations of drugs using nanovectors are available in the literature [4,5]. The reported information suggests the potential advantages of this formulative approach. As a matter of fact, the administration of retinoic acid and betamethasone in deformable liposomes not only improved the skin penetration of both compounds but also ameliorated their synergistic effects [4]. The combination of ropivacaine and meloxicam in nanostructured lipid carriers enhanced the skin penetration of both drugs with respect to a conventional formulation containing the free drugs [5].

The permeation of LD has been deeply investigated, and lipid-based carriers have been tested with the aim of prolonging the duration of action and the analgesic effect rather than improving skin permeation [6,7]. Conversely, the poor skin permeation data available for CBD indicate that the crossing through the skin of this compound is limited by its high hydrophobicity and affinity for stratum corneum components. Indeed, the interactions with ceramides favor the retention of CBD in the superficial skin layers rather than the penetration in the dermis [8]. However, the encapsulation of CBD in ethosomal carriers demonstrated an improvement of the permeation properties of CBD, allowing the delivery of the drug in the systemic circulation after administration in mice [9].

Recently, we proposed a novel liposomal carrier characterized by the encapsulation of micelles in the aqueous core of deformable liposomes named Drug-in-Micelles-in-Liposomes system, DiMiL [10]. DiMiL presents the advantage to deliver the loaded drugs in the deeper skin layers; therefore, it can represent a good solution to ameliorate the limited skin biopharmaceutical properties of CBD. Furthermore, due to its dual-carrier features, DiMiL can accommodate two hydrophobic drugs in different compartments, one in the micelles and the other in the lipid bilayer, avoiding possible competition during the loading steps.

To demonstrate the feasibility and possible advantages of the CBD/LD fixed combination in a deformable liposome, we compare the performances of two formulations prepared by a gradient transmembrane method or DiMiL approach. Both approaches were designed to load drugs in the two liposomal compartments, i.e., the inner core and the bilayer. The compound (CBD or LD) to be loaded in the membrane was chosen on the basis of the affinity for phospholipids determined by differential scanning calorimetry (DSC) since the higher the affinity for the membrane, the lower the risk of drug leakage. Furthermore, since the presence of a surfactant in the bilayer is foreseen and information on its effect on fixed combinations intended for a cutaneous administration is not available, the effect of this functional excipient on the performances of the fixed combination formulations, in terms of deformability, drug release, and skin penetration were also studied.

## 2. Materials and Methods

### 2.1. Materials

Soy-phosphatidylcholine (S 100) and 1,2-dipalmitoyl-sn-glycero-3-phosphocholine (DPPC) were kindly provided by Lipoid (Steinhausen, Switzerland); Kolliphor HS 15 (K-HS15) was provided by BASF (Cesano Maderno, Italy); LD base was obtained from A.C.E.F. S.p.A. (Fiorenzuola d’Arda, Italy) and Tween^®^ 80 from Croda (Chocques, France); PEG 400 and ascorbic acid were purchased from VWR (VWR Prolabo Chemicals, Leuven, Belgium), whereas ammonium molybdate, sodium dihydrogen phosphate, and analytical-grade solvents were obtained from Merck Life Science (Milan, Italy); CBD was kindly gifted by Indena S.p.A. (Milano, Italy). Versatis^®^ (LD Plaster) was from Grunenthal (Aachen, Germany). Finally, sodium hydroxide and orthophosphoric acid were obtained from Carlo Erba (Milan, Italy).

### 2.2. Methods

#### 2.2.1. Differential Scanning Calorimetry (DSC) Studies

The relative affinity of LD and CBD with the lipid bilayer was studied by DSC, monitoring the trend of the phase transitions of the model lipid DPPC after interactions with the drugs used in this study. For these studies, multilamellar vesicles of DPPC in water were prepared by the conventional thin lipid film hydration method (as described below) and loaded with increasing amounts of LD and CBD (from 0 to 0.5 mg/mL).

Thirty microliters of liposomal samples were then transferred to an aluminum pan, sealed, and subjected to cooling from 25 to 0 °C at 1 K min^−1^, kept at 0 °C for 5 min, and then heated to 60 °C at 2 K min^−1^. The DSC cell and refrigerated cooling systems were purged with dry nitrogen at 80 and 120 mL/min, respectively. The system was calibrated using an indium standard. All data were treated with Star^e^ System software Version 10.0 (Mettler Toledo, Milan, Italy).

#### 2.2.2. Preparation of Liposomes

**Figure 1:**
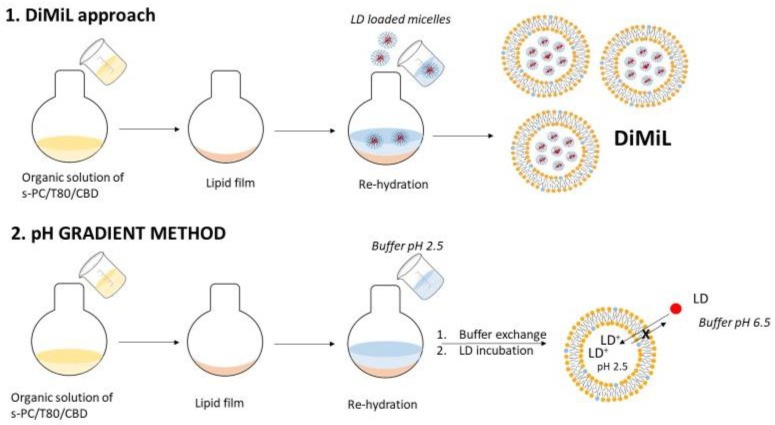
Scheme of DiMiL and G-liposomes preparation methods.

Liposomes were prepared by thin lipid film hydration method. Soy-phosphatidylcholine (sPC) and, when present, Tween 80 and CBD were dissolved in chloroform, and the organic solvent was evaporated under reduced pressures at 40 °C for 1 h using a rotatory evaporator (RII, Buchi, Flawil, Switzerland). As exemplified in Figure 1, the lipid film then was hydrated either with a micellar dispersion (10% *w*/*v* K-HS15) containing LD (for DiMiL systems) or with a buffer at pH 2.5 (in the case of G-liposomes). In all cases, the final lipid concentration was fixed at 30 mg/mL, while sPC and Tween 80 were in 85:15 *w*/*w* ratio. Resulting multilamellar vesicles were downsized by extrusion using a mini-extruder (Avanti Polar Lipids, Alabaster, AL, USA) and passing the samples five times through 200 nm and six times through 100 nm polycarbonate filters (Nuclepore Track-Etched membranes, Whatman, UK).

For the pH gradient method (G), unilamellar vesicles underwent ultrafiltration using Amicon^®^ Ultra-4 (Merck Millipore Ltd., Tullagreen, Ireland) to exchange the external buffer (replacing the buffer at pH 2.5 with a phosphate buffer at pH 6.5). The LD base was then dissolved in the same buffer in order to be loaded in preformed empty vesicles by exploiting the pH gradient formed between the inner core of liposomes and the dispersant medium. In particular, after a preliminary evaluation, an aliquot of 0.670 mL of LD solution was added dropwise to 1 mL of empty unilamellar liposomes previously prepared (Figure 1). The mixture was stirred for three hours at 37 °C.

Final DiMiL and liposomes formulations were purified from unentrapped materials by ultrafiltration. Vesicles remaining at the top of the tube were re-dispersed to the starting volume and assayed for drug contents, as described below.

#### 2.2.3. Physico-Chemical Characterization of Liposomes

DiMiL and pH-gradient liposomes were characterized in terms of particle size distributions by Dynamic Light Scattering (DLS) using a Zetasizer Nano ZS (Malvern Instruments, Malvern, UK). Samples were inserted in a disposable cuvette after a 1:10 dilution in 0.22 µm filtered milliQ^®^ water and analyses were carried out at 25 °C with a detection angle of 173°. Three measurements were taken for each sample, and the results are expressed as the mean and standard deviation. Lipid concentrations were instead assessed by a modified Rouser method [11].

Vesicles deformability was studied by using an in-house protocol [12]. Briefly, 1 mL of G or DiMiL samples (at a lipid concentration of 0.23 mM) was loaded in a 1 mL glass syringe, inserted in the mini-extruder holder containing a polycarbonate membrane with pores of 50 nm. The syringe plunger was then moved at a constant rate of 1 mm/s using a dynamometer (INSTRON^®^ 5965, ITW Test and Measurement Italia S.r.l., Turin, Italy), thus forcing the liposomes through the membrane’s pores. The force (N) required to move the syringe plunger was plotted against the plunger’s displacement (mm), and the slope of this plot, namely, the constant of deformability (k), was derived. The higher the k value, the lower the deformability of the carriers. At the end of the test, the intact vesicle’s concentration in the extruded dispersion was also measured by Nanoparticle Tracking Analysis (NTA) using a Nanosight NS 300 (Malvern Instrument, Malvern, UK).

Encapsulation efficiency was determined after breaking the vesicles through a 1:100 dilution in methanol to release the free drug. LD and CBD were quantified by HPLC using an HP 1100 ChemStation system (Agilent Technologies, Inc., Santa Clara, CA, USA). A mixture of acetonitrile/acidified water at pH 2.5 (88/12 *v*/*v*) was used as the mobile phase. The flow rate was fixed at 1.0 mL/min. The injection volume was 20 µL. The UV detector was set at a wavelength of 215 nm. A reverse-phase C8 column (InertClone™, 5 µm, 150 × 4.6 mm; Phenomenex, Inc., Torrance, CA, USA) was used. The retention time of LD in such conditions was 3 min, whereas that of CBD was 14 min. Calibration curves for both drugs were built in the range of 0.5–100 µg/mL. Encapsulation efficiencies were provided by the percentage ratio between the amount of drug found in the liposomes and the total amount of drug loaded at the beginning of the preparation.

#### 2.2.4. In Vitro Drug Release Studies

In vitro drug release studies were performed using Franz diffusion cells possessing a receiving volume of about 3.0 mL and a surface area of 0.636 cm^2^. An inert synthetic membrane of regenerated cellulose (Cuprophan^®^, Millipore HAWP) was used to follow the release of LD from the formulations, whereas a nitrate cellulose membrane (Sartorius Stedim Biotech GmbH, Göttingen, Germany) was used in the case of CBD because of the interaction between CBD and Cuprophan, which limited the diffusion of free CBD through the membrane, as verified in preliminary assays carried out on a CBD control solution. Three hundred microliters of each formulation were loaded in the donor phase, whereas a 2.5% *w*/*v* K-HS15 dispersion was used as the release medium to assure sink conditions. Donor and receptor compartments were sealed together by means of a clamp.

The medium was maintained under stirring and at a controlled temperature of 37 ± 1 °C by means of a circulating water bath. At fixed time intervals (0.15, 0.30, 1, 1.5, 2, 3, 4, 7, and 24 h), 200 μL of release medium was withdrawn and replaced with an equal volume of fresh medium. The amount of LD and CBD at each time were assayed using the analytical method detailed in the previous section.

#### 2.2.5. In Vitro Skin Permeability Studies

The in vitro permeability studies were performed under non-occlusive conditions by using modified Franz diffusion cells and porcine ear skin as a membrane.

Full-thickness porcine ear skin was initially cleaned with tap water. The hairs were removed and cut in samples with a thickness of 0.74 ± 0.12 mm by using a dermatome (The Soutter Medical, Casorate Primo, Italy).

Skin samples were mounted on Franz diffusion cells with the stratum corneum facing the donor compartment.

At the beginning of the permeation experiment, tested formulations were diluted with ultrapure water at the same drug concentration (415.1 µg/cm^2^ for LD and 358 µg/cm^2^ for CBD), and 300 µL was loaded in the donor chamber of the Franz’s cell. Receptor compartment was filled with a degassed dispersion of 2.5% *w*/*v* K-HS15, previously filtered with 0.22 µm nylon filter. Franz diffusion cells were assembled as described above, and the temperature was monitored using a circulating bath to reach a temperature of 32 ± 1 °C at the membrane’s surface. At predetermined times (1, 3, 5, 7, and 24 h), 200 μL of samples was withdrawn from the receiver compartment and replaced with the same volume of fresh receiver medium. Sink conditions were maintained throughout the experiments.

The cumulative amount of CBD and LD permeated through the skin per unit area (QP) was calculated from drug concentrations in the receiving medium and plotted as a function of time. The steady-state flux (J) was determined as the slope of the linear portion of the plot. Lag time was determined as the x-intercept of the slope at a steady state. The obtained results were expressed as an average of parallel experiments performed at least in triplicate.

An LD plaster (Versatis) was used as a positive control. This medicinal product was selected since its main indication is the local treatment of neuropathic pain. In the case of CBD, a 1% CBD solution using PEG 400 as solvent was chosen as a reference since no topical medicinal products were available on the market.

At the end of permeation experiments, skin samples were removed from the Franz diffusion cells, and each side was gently washed with 5 mL of methanol to eliminate the unabsorbed drug. Subsequently, the epidermis was mechanically separated from the dermis by using tweezers after heating the sample at 100 °C for 30 s. The dermal and epidermal layers were dried, weighted, thinly sliced, and placed in 3 and 2 mL of fresh methanol, respectively. Each sample was directly sonicated using a probe sonicator for 15 min (30 s on/10 s off, 70% amplitude) to extract drugs retained in the tissue. Finally, each sample underwent centrifugation for 10 min at 3000 g, and the supernatant was filtered (0.45 μm PP syringe filter) and analyzed by HPLC.

## 3. Results and Discussion

### 3.1. Physico-Chemical Characterization of Liposomes

**Figure 2:**
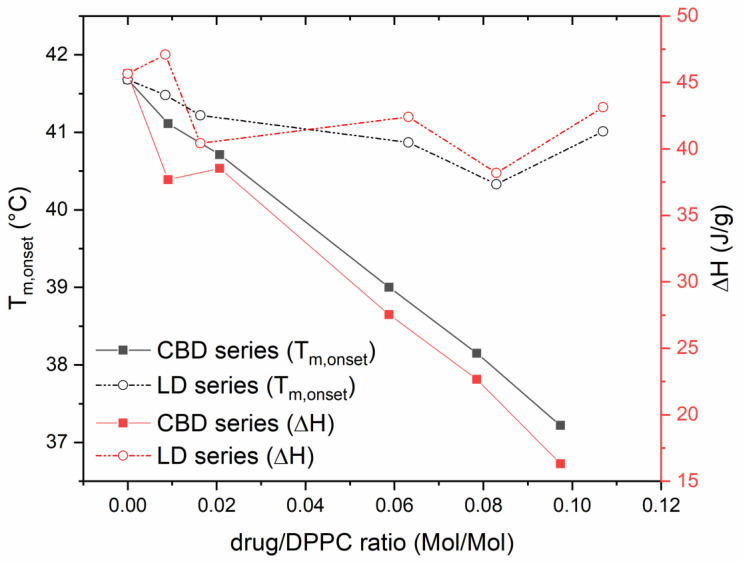
Trends of main transition temperature onset (T_m,onset_) and transition enthalpy (ΔH) of DPPC as a function of CBD or LD/lipid molar ratio. The coefficient of variation in the presented data was lower at 0.5% for the T_m,onset_ and lower than 2% for ΔH.

The addition of CBD to DPPC membrane caused a linear, concentration-dependent depression of both the main transition (T_m_) onset and transition enthalpy of DPPC (Figure 2). These data suggest that CBD has a strong affinity for lipids, and it is able to strongly intercalate in the lipid bilayer, causing the suppression of the cohesive interactions between adjacent phospholipid molecules. Instead, LD did not exert a relevant effect on both parameters considered over all ranges of the investigated molar ratio (Figure 2). Based on these results, CBD was selected to be loaded in the liposome bilayer because the stronger interaction with lipid moieties may assure a more stable encapsulation of the drug. It is also worth noting that according to DSC data, CBD is able to act as a fluidizing agent after encapsulation in the liposome bilayer. To further understand the contribution of CBD to the overall deformability of the liposomes, DiMiL and G-liposomes were prepared with and without Tween 80. Accordingly, all prepared liposomes resulted very high deformable (constant of deformability, *k*, very close to zero) regardless of the presence of the edge activator (Tween 80) in the bilayer. In the case of formulations prepared by gradient methods, the addition of Tween 80 further increases the deformability of the carrier (Table 1). On the other hand, comparing the two formulations without the polysorbate, DiMiL(-), and G(-), the DiMiL system resulted to have a higher flexibility, and this can be ascribed to the partial distribution of kolliphor micelles in the bilayer during the formation of the vesicle, as already suggested in our previous work [10]. Since K-HS15 is a surfactant with an HLB value similar to that of Tween 80, it may be hypothesized that it also acts as edge activator. This hypothesis should be verified in specific studies that are, however, outside of the aim of the present study.

The prepared liposomes showed a mean diameter around 100 nm with a good polydispersion index (lower than 0.1 in all cases) and allowed a quite high encapsulation of both drugs (Table 1).

Two different approaches have been used to load LD in the aqueous core: the DiMiL approach, which relies on the encapsulation of the LD base itself through micelle entrapment, and an active loading method, which instead relies on the encapsulation of the weak base in preformed vesicles by exploiting a pH transmembrane gradient (Figure 1). The LD base is a suitable candidate for this transmembrane method, possessing a logP of 2.3 and a pKa of 7.9 [13]. Preformed vesicles were formed with the inner core at a pH of 2.5, whereas the external medium was maintained at pH 6.5 using a phosphate buffer, reaching a satisfactory encapsulation efficiency (Table 1). However, the presence of Tween 80 significantly reduced the loading efficiency, and this happened by using both DiMiL and transmembrane approaches. In the case of G formulations, the data can be justified by the competition between LD and Tween 80 during the (trans)membrane diffusion of the LD base towards the inner core of liposomes. In the case of DiMiL formulations, since it was demonstrated that a partial distribution of micelles in the bilayer occurs during vesicle formations [10], the decrease in encapsulation efficiency can depend on a partial exit of LD instead during the exchange between Kolliphor micelles and Tween 80 during the hydration process.

As expected according to DSC data, the encapsulation efficiency was higher for CBD, and in this case, the presence of Tween 80 did not seem to induce any influences. On the contrary, in the case of G formulations, the transmembrane passage of the LD during the active loading process caused the partial expulsion of CBD from the bilayer, resulting in a decrease in the overall encapsulation efficiency of CBD (Table 1).

### 3.2. In Vitro Drug Release and Skin Permeability Studies

**Figure 3:**
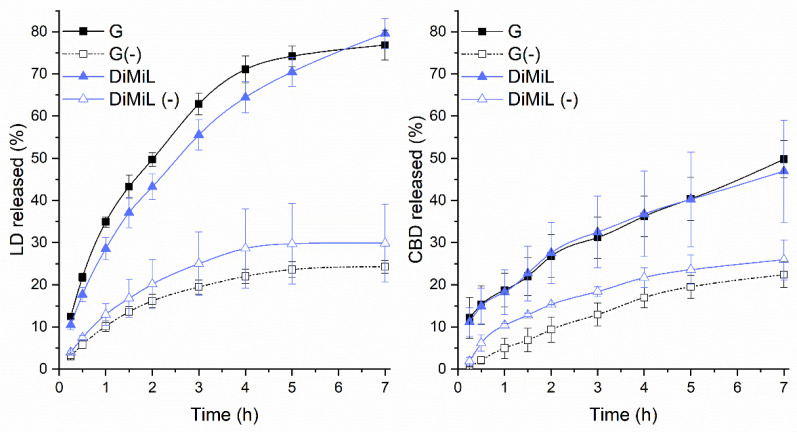
In vitro release profile of LD and CBD from liposome-based formulations in the study.

The in vitro drug release profile of both drugs was superimposable in the case of DiMiL/G and DiMiL(-)/G(-) couples, suggesting that Tween 80 exerts a key role in determining the extent of drug diffusion through the membrane (Figure 3). In both cases, the lack of Tween 80 led to a more controlled and sustained drug release, probably because of the higher packing of the membrane. In fact, although all formulations resulted highly deformable in vitro deformability assays, the constant of deformability, k, increased of about ten times in the formulations with Tween 80 with respect to Tween 80-free ones. A similar drug release profile found for G/DiMiL couple was expected in the case of CBD as it sits in the liposomal membrane, but it was surprising in the case of LD. According to these data, in fact, LD’s release rates were comparable when the drug is encapsulated in the aqueous core of liposomes either as a base in the micelles and as salts, and the drug diffusion rate was mainly governed by the structure of the bilayer in the presence or absence of Tween 80. Nevertheless, it is important to underline that the release medium selected to assure sink conditions has a pH of 6.5, and then the equilibrium during the in vitro release test shifted towards the non-ionized form that is more easily released from the inner core of liposomes.

**Figure 4:**
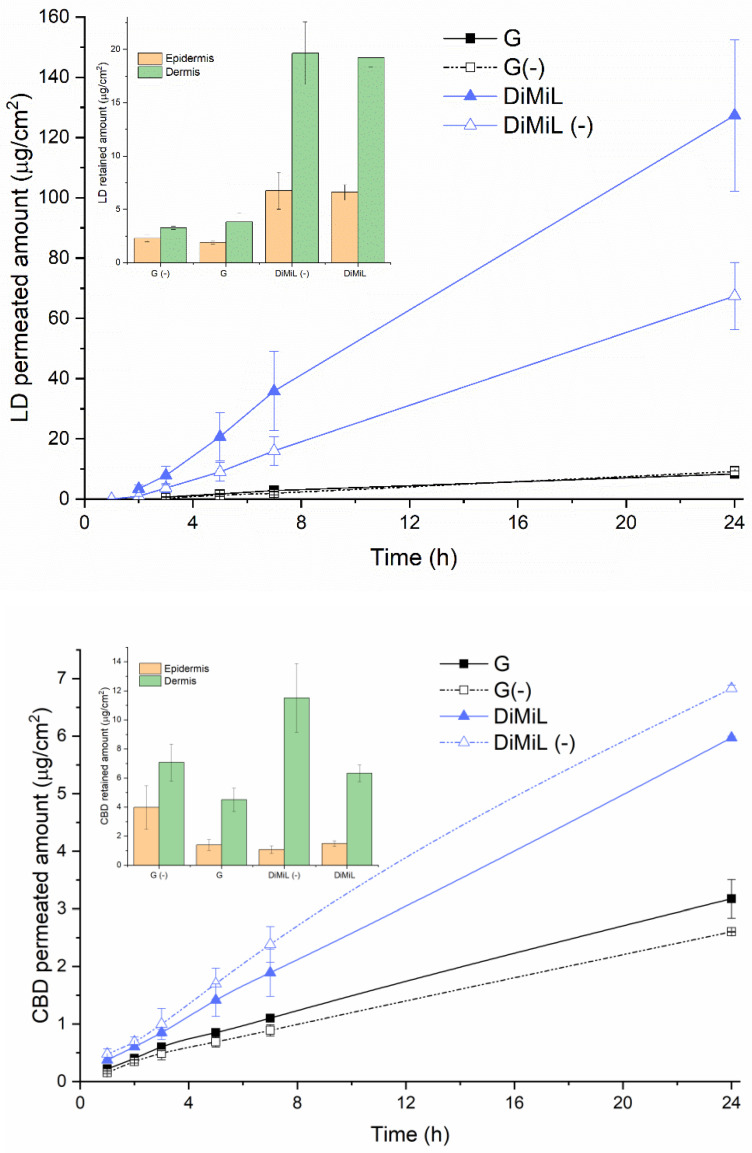
Penetration pattern of LD and CBD loaded in G-liposomes and DiMiL systems.

Moving the attention to skin-penetration studies, DiMiL was able to deliver both drugs in the deeper skin layers, leading to an increase in the permeation of CBD and LD through porcine ear skin when compared to G-liposomes (Figure 4).

In the case of LD, it was already reported that sPC-based deformable liposomes were able to enhance the permeation of LD with respect to plain-drug solutions [14]. In this study, it was demonstrated that the DiMiL system is even more efficient than conventional deformable liposomes. As shown in Figure 4, DiMiL significantly reduced the lag time of drug permeation (that is desirable for fasting the onset of action) and improved the flux of the drug of about one order of magnitude (G: 0.28 ± 0.16 µg/cm^2^/h; DiMiL: 5.62 ± 2.42 µg/cm^2^/h). As mentioned before for drug release, this result is surely affected by the form of LD since it is well known that the LD base diffuses better through the skin compared to the ionized form [15]. It is worth noting that the permeation pattern of LD base itself was higher in the presence of Tween 80 in the bilayer (Figure 4), confirming that in addition to deformability properties, the entire organization of the DiMiL system is responsible for the penetration behavior of this novel carrier, as already demonstrated with other model drugs [10]. Along with increased permeability, DiMiL favored the accumulation of both drugs in the dermis to be available for the targeting of the receptors located in the nerve fibers for deep pain relief [3]. It is interesting to observe that the contribution of Tween 80 on the extent of drug retention in dermis was drug dependent; in particular, in the case of CBD, the edge activator seemed to have a suppressive effect (Figure 4). This behavior is in line with the data recently published by Junaid and co-workers that also observed a decrease in the dermis accumulation of CBD in the presence of some penetration enhancers [16], and it is worth of further investigation to observe if the presence of Tween 80 would favor the permeation of CBD in the receiver compartment without being retained in the dermal layer. Besides the effect of Tween 80, the localization of both drugs in the dermis rather than epidermis regardless of the relative physico-chemical properties (CBD has a poor tendency to distribute beyond the stratum corneum), suggests again that DiMiL acts as a real carrier for loaded drugs and not as a penetration enhancer, as previously hypothesized [10]. Finally, it is important to highlight that the extent of drugs that reached the deep skin was significant, also considering the poor drug concentration in liposome-based formulations (i.e., only 0.2% *w*/*v* compared to 5–10% and 2–5% present in commercial available CBD and LD topical formulations, respectively). This is an important achievement because of the maximization of the effect with a decrease in the toxic dose of drugs that could reach systemic circulation.

It should also be underlined that all formulations are more efficient with respect to the two selected controls, i.e., the LD plaster and the CBD PEG 400 solution, despite of the larger LD and CBD amounts present in the control formulations (Table 2). This difference in terms of concentration was desired mainly in the case of CBD to point out that a good permeation pattern of the drug can be achieved also at low doses. In fact, DiMiL’s formulation not only significantly increased the drug’s flux (that was one order of magnitude higher for DiMiL system) but also led to the abolishment of lag times (that was instead 3 ± 1 h in the case of the control formulation). In the case of LD, the flux obtained using the plaster was slightly higher but not significantly different from that obtained using DiMiL; however, a 10-times-higher drug dosage was required.

## 4. Conclusions

The request of advanced formulations for fixed-dose combinations that ameliorate the risk–benefit effect is increasing not only in the case of systemic pathologies but also for the treatment of cutaneous pathologies.

The data presented in this study revealed that the DiMiL system can contain the drug in the dermis more efficiently with respect to the formulations prepared by the pH gradient system. Furthermore, and more interestingly in the case of DiMiL(-) formulations, the ratio between the retained amounts (µg/cm^2^) of LD and CBD extracted in the dermis at the end of each experiment was close to one (0.97 ± 0.4), namely, the same LD/CBD ratio present in liposomes, suggesting that the skin penetration profile of the cargoes is driven by the liposome’s formulation. In fact, all other compositions in study did not allow the retainment of a fixed drug amount in the skin. These data, if confirmed, can open new scenarios in the design of topically fixed combinations, since nanocarriers can play an important role in tuning the ratio in drug concentrations in the different skin layers, as in other administration routes. Furthermore, the skin permeation data compared with reference formulations containing the free drug revealed that deformable liposomes are more efficient, complying with regulatory requirements of reducing the administration dose. Altogether the evidence derived from this work can open a path for the design of nanocarrier-based products for fixed drug combinations to deal with unmet medical needs.

## Figures and Tables

**Table 1:** Main composition and physico-chemical features of G and DiMiL formulations.

Form.	Lipid Bilayer		Aqueous Phase		Physico-Chemical Characteristics			
	Tween 80 (% *w*/*w*)	sPC (% *w*/*w*)	Inner Core	Dispersant Medium	EE%		d (nm)	Deformability K (N/mm)
					CBD	LD		
DiMiL	15	85	K-HS15 5 *w*/*v*%	Depurated water	92.2 ± 3.4	59.3 ± 0.3	81.3 ± 0.2	0.001 ± 0.000
DiMiL(-)	-	100	K-HS15 5 *w*/*v*%	Depurated water	94.0 ± 5.0	74.8 ± 4.6	96.0 ± 0.6	0.018 ± 0.007
G*	15	85	Buffer pH 2.5	Buffer pH 6.5	63.7 ± 4.1	53.9 ± 3.0	107.1 ± 0.7	0.003 ± 0.001
G(-)	-	100	Buffer pH 2.5	Buffer pH 6.5	76.0 ± 1.4	88.9 ± 2.5	103.0 ± 0.2	0.040 ± 0.009

G*: gradient method; the minus sign stands for “*without Tween 80*”.

**Table 2:** Skin permeation fluxes obtained using the control and tested LD and CBD formulations.

Formulation	Steady-State Flux (μg/cm^2^/h)	
	LD	CBD
Versatis lidocaine plaster *	7.51 ± 1.27	-
1% CBD PEG 400 solution **	-	0.03 ± 0.01
G(-)	0.35 ± 0.06	0.07 ± 0.02
G	0.28 ± 0.16	0.12 ± 0.03
DIMIL(-)	3.00 ± 0.82	0.19 ± 0.10
DIMIL	5.62 ± 2.42	0.10 ± 0.07

* Applied dose: 5 mg/cm^2^; ** applied dose: 4.7 mg/cm^2^.
